# Delayed Laparoscopic Cholecystectomy Is Safe and Effective for Acute Severe Calculous Cholecystitis in Patients with Advanced Cirrhosis: A Single Center Experience

**DOI:** 10.1155/2014/178908

**Published:** 2014-03-19

**Authors:** Zhicheng Yao, Kunpeng Hu, Pingzhu Huang, He Huang, Xingui Chen, Peisheng Yang, Bo Liu

**Affiliations:** Department of General Surgery, Lingnan Hospital of Sun Yat-sen University, Kaichuang Road No. 2693, Luogang District, Guangzhou City, Guangdong Province 510530, China

## Abstract

Acute calculous cholecystitis is a common disease in cirrhotic patients. Laparoscopic cholecystectomy can resolve this problem but is performed based on the premise that the local inflammation must been controlled. An Initial ultrasound guided percutaneous transhepatic cholecystostomy may reduce the local inflammation and provide advantages in subsequent surgery. In this paper, we detailed our experience of treating acute severe calculous cholecystitis in patients with advanced cirrhosis by delayed laparoscopic cholecystectomy plus initiated ultrasound guided percutaneous transhepatic cholecystostomy and provided the analysis of the treatment effect. We hope this paper can provided a kind of standard procedure for this special disease; however, further prospective comparative randomized trials are needed to assess this treatment in cirrhotic patients with acute cholecystitis.

## 1. Introduction

Cholelithiasis is a common disease in cirrhotic patients. According to some reports, the mortality rate in this patient subset is nearly twice that of noncirrhotic patients [1, 2]. An incarcerated gallbladder stone in the gallbladder neck may result in acute cholecystitis (AC), a finding that is also commonly observed in cirrhotic patients, with subsequent distension and severe bacterial inflammation of the gallbladder [3].

The gold standard treatment for acute cholecystitis is laparoscopic cholecystectomy (LC) [4]. A large body of research has demonstrated that LC in patients with early, well-compensated cirrhosis is safe and offers advantages over the open approach [5–7]. However, the cirrhotic background and severe inflammation may prolong the operative time and increase both blood loss and the conversion rate to an open procedure. Thus, immediate LC is often a difficult and risky procedure in this patient group.

Gallbladder aspiration, introduced in 1985 as a diagnostic procedure, is becoming a treatment option for patients with AC and other high-risk conditions [8]. The technique of sonographically guided percutaneous transhepatic cholecystostomy (PTC), which results in gallbladder decompression, has been demonstrated as safe and effective in many studies [9, 10]. After such a procedure, the local inflammation can be controlled, thereby allowing a delayed LC, which has been simplified and become a safer procedure in recent years [11]. Nevertheless, few published articles have addressed the issue of delayed LC in patients with advanced cirrhosis. In this retrospective study, we aimed to evaluate the outcomes of patients with liver cirrhosis who underwent delayed LC for incarcerated gallbladder disease at our institution during the last 7 years.

## 2. Patients and Methods

### 2.1. Patients

Between July 2004 and September 2011, 29 patients with acute cholecystitis caused by an incarcerated stone in the neck of gallbladder and who were evaluated in the Department of General Surgery at the 3rd Affiliated Hospital of Sun Yat-sen University in Guangzhou, China were enrolled in the study. All patients with acute cholecystitis were clinically diagnosed by right upper abdominal quadrant pain, significantly elevated white blood cell counts, and a positive Murphy's sign. All patients were diagnosed with hepatitis B virus- (HBV-) related liver cirrhosis. In each patient, the diagnosis was confirmed with the ultrasonographic findings of gallbladder distention, a thickened gallbladder wall, and an incarcerated stone in the neck of the gallbladder. The liver function status in all patients was determined by the Child-Pugh classification. The diagnosis of cirrhosis was established by the ultrasound (US) findings and by liver biopsy performed during surgery.

### 2.2. PTC

The following procedural steps were performed in PTC. First, the gallbladder was examined by US to determine the most suitable puncture point. Following administration of local anesthesia, a Seldinger needle was inserted percutaneously into the tumid gallbladder with passage through the hepatic parenchyma performed by an interventional radiologist under US guidance. The sample of gallbladder fluid drained from the needle was collected for bacteriologic studies, and an 8.5-F pigtail catheter was then placed under US guidance using a two-step method that involved guidewire exchange. A sterile bag was connected to the catheter for continuous drainage.

### 2.3. LC

A standard LC approach was performed using a 4-trocar technique. All gallbladders were removed* in toto*, and in no case was subtotal cholecystectomy performed. Intraoperative cholangiography was only selectively performed. The specimens were sent for routine pathological examination. To avoid damage to the paraumbilical veins, a routine US examination was performed preoperatively, and the first trocar was placed using the Hasson technique. A lower pressure of carbon dioxide pneumoperitoneum was used to lessen further damage to hepatic function. Harmonic ACE shears (Ethicon Endo-Surgery, Cincinnati, OH) were used to separate the gallbladder during surgery. The preoperative preparations included the procurement of adequate fresh frozen plasma and cryoprecipitate, and transfusion was implemented to improve the coagulation status, as necessary.

### 2.4. Data Presentation

Categorical values were expressed as percentages, with continuous variables expressed as the mean ± standard deviation.

## 3. Results

The study included 17 men and 12 women (mean age, 51.3 ± 8.1 years; range, 44–71 years). Clinically, all patients presented with pain (epigastric or in the right hypochondrium), 17 (58.6%) were febrile, 11 (37.9%) had vomiting, and 5 (17.2%) were jaundiced. Ultrasound revealed a thickened gallbladder wall in all patients (mean thickness, 6.2 ± 1.1 mm). All gallbladders were enlarged. Twenty-one patients (72.4%) demonstrated an incarcerated stone in the neck of the gallbladder (by US), two patients (6.9%) were negative for this finding, and an indistinct stone was noted in six patients (20.7%) (see Table 1).

After the diagnosis of acute cholecystitis was established, all patients were treated by US-guided PTC. The symptoms of all patients resolved within three days after PTC, and all tubes were correctly placed, according to the intraoperative findings. One patient (5.6%) had puncture-related complications and developed hemorrhage, which was drained via the catheter and spontaneously resolved, and 11 patients (37.9%) presented with pain at the catheter site. None of the patients developed bile leakage, and none of the catheters became dislodged.

After the patients were admitted to the hospital, an empirical course of antibiotics was started and later adjusted based on the results of bacterial cultures. These cultures revealed* Escherichia coli* in nine cases (31%),* Klebsiella pneumoniae* in four cases (13.8%), and* S. aureus* in two cases (6.9%). Fourteen patients (48.3%) had negative cultures.

The mean drainage treatment period was 8.1 ± 1.4 days (range, 7–12 days). All patients agreed to LC for total gallbladder resection, and no cases were converted to an open procedure. The mean operative time was 51.5 ± 11.8 min, and the mean bleeding volume was 45.8 ± 7.1 mL. The mean hospitalization time for the 29 patients was 15 ± 7.2 days (range, 10–41 days), and the average hospitalization cost was $3,166 USD. The intraoperative and postoperative morbidity data are presented in Table 2. Postoperative deterioration in liver function tests was noted at 48 hours in 4 patients (13.8%); these values invariably recovered to baseline levels within 2 weeks. Worsening of ascites was observed in only 1 patient (3.45%), and 5 patients were transfused with fresh frozen plasma (17.2%). None of patients died during hospitalization.

## 4. Discussion

Gallstones invariably occur in the gallbladders of cirrhotic individuals. Some studies have pointed to increased intravascular hemolysis, hypersplenism, and increased estrogen levels that are associated with reduced gallbladder emptying and motility as causes; increased estrogen levels may represent the main cause [12, 13]. The presence of a stone that is incarcerated in the neck of gallbladder represents one type of cholelithiasis that may readily lead to AC. This type of cholelithiasis fulfills the three conditions involved in the development of AC (increased intraluminal pressure, chemical injury of the mucosa from bile salts, and bacterial infection). Therefore, AC in patients with cirrhosis is not a rare occurrence in gastroenterology clinic.

Cholecystectomy, including open and laparoscopic surgery, is a standard treatment for AC. Many studies have demonstrated that open cholecystectomy in cirrhotic patients is associated with high mortality and morbidity rates [14–16]. Most complications and deaths have been related to gallbladder bed bleeding, postoperative liver failure, and systemic infection.

The LC technique was developed as a treatment option in the 1980s; however, since the introduction of LC, cirrhosis has been viewed as an absolute or relative contraindication for LC because of the potential risks of bleeding and liver failure [17, 18]. Even published guidelines have stated that liver cirrhosis is a contraindication to LC [19]. However, in recent years, LC has evolved as a safe and well-tolerated procedure in selecting cirrhotic patients because of increased clinical experience and instrumentation improvements. A national study that involved 3,482 patients demonstrated that LC should be the preferred initial approach in cirrhotic patients [20]. Cirrhotic patients can benefit from the less extensive operative wound left by LC, which may result in shorter hospital stays, faster postoperative rehabilitation, and a reduced incidence of wound complications compared with open cholecystectomy. Many studies have indicated satisfactory results for LC in selecting groups of cirrhotic patients. In a small retrospective study, Leandros and coworkers [21] observed that LC could be performed reasonably safely and with acceptable morbidity in patients with well-compensated liver function. A well-designed, prospective randomized study also demonstrated that LC is a preferred choice for cirrhotic patients compared to open cholecystectomy [22]. Two meta-analyses also concluded that the laparoscopic approach might offer advantages of less blood loss, shorter operative time, and shorter length of hospitalization in patients with cirrhosis. However, note that the patients in these studies were primarily Child-Pugh class A or B, with few Child-Pugh class C patients. Therefore, according to these studies, it is difficult to conclude that LC is a safe treatment option for Child-Pugh class C patients [23, 24].

Percutaneous transhepatic cholecystostomy (PTC) is a convenient and safe treatment method for AC that can be performed successfully with few complications. PTC is an appropriate choice for patients who develop AC and who have poor prognoses (Child-Pugh class C) [25]. Successful performance of PTC readily relieves symptoms within a brief time period. This effectiveness appears because of the rapid reduction of pressure within the gallbladder, which is induced by PTC. The reduction of intraluminal pressure is a critical goal of therapy that may lead to complete resolution of the clinical syndrome in many cases, even in those cases involving infected bile [26]. The most common delayed complication (37.9%) was catheter site pain, which was resolved by small doses of analgesics. During hospitalization, the meticulous care of the cholecystostomy tubes is of utmost importance. Measures to prevent catheter loss include secure fixation to the skin and the use of self-retaining loop catheters to help minimize the risk of dislodgment.

Medical treatment decisions for cirrhotic patients with acute cholecystitis caused by an incarcerated gallbladder stone can be problematic. Although the severity of inflammation presents a limiting factor to immediate surgery, unacceptably high rates of morbidity and mortality remain in this patient group [27]. PTC is a safe and conservative procedure that can be performed without removing the gallbladder; reported rates of acute cholecystitis recurrence after cholecystostomy in patients with gallstones can be as high as 25% to 30% [28]. Therefore, the gallbladder pathology may represent a potential source of recurrence after discharge.

All of our patients who underwent PTC demonstrated clinical improvement within 3 days; this improvement helped these patients transition to acceptable surgical conditions. In these patients, gallbladder resection that is performed completely by laparoscopy may present risks in situations involving tenacious posterior wall separation, which may lead to uncontrolled bleeding. In this situation, laparoscopic subtotal cholecystectomy presents a better choice [29]. To avoid severe complications, an appropriate modification of subtotal cholecystectomy should be considered, which will depend on the risks attendant to the individual situation [30]. Note that we achieved a complete LC without modifications in all study patients because the PTC reduced the local inflammation, thereby diminishing the degree of operative difficulty.

Conversion to an open procedure is another issue in treating AC in cirrhotic patients, with conversion rates during LC that have ranged from 0% to 9% [31, 32]. Our conversion rate to open cholecystectomy (0%) was the lowest that has been reported in the published data for LC and may have been because of the effectiveness of PTC in reducing the local inflammation. While conversion is not considered a complication, it can increase the patients' medical and economic burdens. Thus, lower conversion rates may also prevent more serious complications.

The incidences of both intraoperative and postoperative morbidity were minor and infrequent. The delayed LC can aid in reducing risks of surgery in cirrhotic patients. The mean operation time was 51.5 min (i.e., shorter than that cited in published reports) [22]. Because a prolonged operative time is frequently associated with increased complication rates [33], the shorter operative time is meaningful in terms of decreased morbidity in a cirrhotic patient population.

## 5. Conclusions

In our experience, initial PTC followed by a delayed LC is a safe and effective procedure for treating cirrhotic patients who present with acute cholecystitis. This sequence achieves resection of the lesion in cirrhotic patients but with minimal morbidity. Further prospective comparative randomized trials are needed to assess this treatment in cirrhotic patients with acute cholecystitis.

## Figures and Tables

**Table 1:** Preoperative patient characteristics.

Category	Clinical data
Sex	29
Male	17
Female	12
Age (years)	51.3 ± 8.1
Child-Pugh classification	—
A	6
B	19
C	4
Symptom	—
Abdominal pain	29
Fever	17
Vomiting	11
Jaundice	5
Ultrasound findings^†^	—
Positive	21
Negative	2
Indistinct	6
Cholecystitis (mm)	—
Gallbladder wall thickness	6.2 ± 1.1
Gallbladder wall length	118 ± 26
Gallbladder wall width	65 ± 11

^†^Positive: a highly echogenic mass with an acoustic shadow that was detectable by US in the neck of the gallbladder and that did not move with a change in patient position. Negative: no findings of stones in the neck of the gallbladder. Indistinct: no clearly detectable stones in the neck of the gallbladder because of the viewing angle.

**Table 2:** Intraoperative and postoperative complications.

Complication	*n*	%
Converted to open cholecystectomy	0	0
Gallbladder bed hemorrhage	0	0
Cholecystohepatic triangle hemorrhage	0	0
Fresh frozen plasma transfusion	5	17.2
Infection of port(s)	0	0
Postoperative deterioration in liver function tests	4	13.8
Postoperative worsening of ascites	1	3.45
Bile leakage	0	0
Gallbladder remnant	0	0

## Abbreviations

LC: Laparoscopic cholecystectomy
PTC: Percutaneous transhepatic cholecystostomy
AC: Acute cholecystitis.
